# Efficacy of Fosfomycin and Its Combination With Aminoglycosides in an Experimental Sepsis Model by Carbapenemase-Producing *Klebsiella pneumoniae* Clinical Strains

**DOI:** 10.3389/fmed.2021.615540

**Published:** 2021-03-26

**Authors:** Tania Cebrero-Cangueiro, Gema Labrador-Herrera, Álvaro Pascual, Caridad Díaz, Jesús Rodríguez-Baño, Jerónimo Pachón, José P. del Palacio, María E. Pachón-Ibáñez, M. Carmen Conejo

**Affiliations:** ^1^Clinical Unit of Infectious Diseases, Microbiology, and Preventive Medicine, University Hospital Virgen del Rocío, Seville, Spain; ^2^Institute of Biomedicine of Seville (IBiS), Virgen del Rocío and Virgen Macarena University Hospitals/Consejo Superior de Investigaciones Científicas (CSIC)/University of Seville, Seville, Spain; ^3^Department of Medicine, University of Seville, Seville, Spain; ^4^Clinical Unit of Infectious Diseases, Microbiology, and Preventive Medicine, Virgen Macarena University Hospital, Seville, Spain; ^5^Department of Microbiology, University of Seville, Seville, Spain; ^6^Fundacion Centro de Excelencia en Investigación de Medicamentos Innovadores en Andalucía, MEDINA Foundation, Granada, Spain

**Keywords:** fosfomycin, aminoglycosides, *in vivo*, time kill curves, carbapenemase-producing *Klebsiella pneumoniae*

## Abstract

Carbapenemase-producing *Klebsiella pneumoniae* infections are an increasing global threat with scarce and uncertain treatment options. In this context, combination therapies are often used for these infections. The bactericidal and synergistic activity of fosfomycin plus amikacin and gentamicin was studied trough time–kill assays against four clonally unrelated clinical isolates of carbapenemase-producing *K. pneumoniae*, VIM-1, VIM-1 plus DHA-1, OXA-48 plus CTXM-15, and KPC-3, respectively. The efficacy of antimicrobials that showed synergistic activity *in vitro* against all the carbapenemase-producing *K. pneumoniae* were tested in monotherapy and in combination, in a murine peritoneal sepsis model. *In vitro*, fosfomycin plus amikacin showed synergistic and bactericidal effect against strains producing VIM-1, VIM-1 plus DHA-1, and OXA-48 plus CTX-M-15. Fosfomycin plus gentamicin had *in vitro* synergistic activity against the strain producing KPC-3. *In vivo*, fosfomycin and amikacin and its combination reduced the spleen bacterial concentration compared with controls groups in animals infected by *K. pneumoniae* producing VIM-1 and OXA-48 plus CTX-M-15. Moreover, amikacin alone and its combination with fosfomycin reduced the bacteremia rate against the VIM-1 producer strain. Contrary to the *in vitro* results, no *in vivo* efficacy was found with fosfomycin plus amikacin against the VIM-1 plus DHA-1 producer strain. Finally, fosfomycin plus gentamicin reduced the bacterial concentration in spleen against the KPC-3 producer strain. In conclusion, our results suggest that fosfomycin plus aminoglycosides has a dissimilar efficacy in the treatment of this severe experimental infection, when caused by different carbapenemase-producing *K. pneumoniae* strains. Fosfomycin plus amikacin or plus gentamycin may be useful to treat infections by OXA-48 plus CTX-M-15 or KPC-3 producer strains, respectively.

## Introduction

*Klebsiella pneumoniae* carbapenemase (KPC)-producers (KPC-KP) represent an increasing global threat worldwide and is one of the most important existing pathogens, especially in endemic areas. KPC-KP causes mostly nosocomial infections although they can occur in healthy people. Most common infections among others are urinary tract, pneumonia and bacteraemia (1). Resistance to carbapenems in *K. pneumoniae* is mainly due to the production of carbapenem-hydrolyzing β-lactamases such as the KPC type (Ambler class A), IMP, VIM and NMD types (Ambler class B) and OXA-48 (Ambler class D), and bacteremia due to these strains is associated with higher rates of treatment failure and death (2). Moreover, carbapenemase producers often show co-resistance to the majority of other antimicrobial agents, leaving scarce and uncertain treatment options, such as tigecycline, colistin, and some aminoglycosides (2–4). Then, the increment of infections by carbapenemase-producing strains prompts the search of new therapies for infections caused by them. Also, awareness of the prevalence and incidence of the specific mechanisms of carbapenem resistance within *K. pneumoniae* is crucial in the prevention of their spread and selection of appropriate treatment options (5).

In the search of optimal treatments for infections by these carbapenemase-producing strains, combination among antibiotics is being explored (6, 7), together with newer options like plazomicin, eravacycline and cefiderocol (8). The most commonly *in vitro* active and potentially useful drugs remain ceftazidime-avibactam and newer inhibitors combinations, gentamicin, amikacin, colistin, tigecycline, and fosfomycin (9). Many *in vitro* studies and some *in vivo* studies have investigated the effects of double and triple combinations of drugs with different mechanisms of action (10, 11). Synergistic actions have been convincingly demonstrated for carbapenem-resistant Enterobacteriaceae (particularly *K. pneumoniae*), *Acinetobacter baumannii*, and *Pseudomonas aeruginosa*. In this regard, the study of Erdem et al. showed *in vitro* that double carbapenem antibiotics plus colistin could be a potential alternative to treat colistin and carbapenem-resistant *K. pneumoniae* (7). Another *in vitro* study against multidrug-resistant *K. pneumoniae* producing KPC-2, KPC-3, NDM-1, OXA-48 and VIM-1 carbapenemases points to polymyxin B in combination with minocycline, rifampicin or fosfomycin as potentially therapy of interest (12). Nevertheless, there are only a few *in vivo* experimental studies that evaluate antimicrobials in combination against carbapenemase producers *K. pneumoniae* strains. Hagihara et al., found that meropenem plus amikacin was effective against KPC-, IMP- and OXA-48-producing *K. pneumoniae* infections, except for NDM type carbapenemase producing (13). Also, the combination of meropenem plus colistin was found to be bactericidal against a KPC-producing *K. pneumoniae* experimental osteomyelitis in rabbits (14). Several studies have suggested fosfomycin combination with other antimicrobials to be explored because of its *in vitro* activity. In this regard, Yu et al., demonstrated *in vitro* synergy with fosfomycin plus imipenem, ertapenem and tigecycline against KPC-producing K. pneumoniae (15). Also, fosfomycin plus gentamicin were synergist *in vitro* against KPC-3 producing strains (16). Nevertheless, and although the combination of fosfomycin to antimicrobials has been found to be active *in vitro*, there are not *in vivo* studies to evaluate this therapeutic alternative, except fosfomycin plus colistin combination in experimental osteomyelitis due to KPC-producing *K. pneumoniae*, which achieved a reduction the bacterial bone concentration (17).

The aim of the present study was evaluate the efficacy of the fosfomycin plus amikacin or gentamicin in a murine peritoneal sepsis model using four *K. pneumoniae* clinical strains producers of the currently most prevalent carbapenemases.

## Materials and Methods

### Bacterial Strains

In this study, we tested four genetically unrelated clinical isolates of KPC-KP: a VIM-1 ST 1603 clone producing isolate, a VIM-1 with the acquired AmpC type beta-lactamase DHA-1 ST 11 producing isolate, a OXA-48 ST 11 clone with the extended spectrum beta-lactemase (ESBL) CTX-M-15, and a KPC-3 ST 512 clone with the broad spectrum beta-lactamases TEM-1 and SHV-11 (18). The identification of the isolates, the presence of carbapenemase genes, and genes coding for other beta-lactamases and the absence of genetic relatedness among the isolates was confirmed by a Microflex LT- MALDI Biotyper mass spectrometer, PCR and sequencing, and PFGE analysis, respectively, as described previously (18). Two of the strains, KPC-3 and VIM-1 (DHA-1) were multidrug-resistant while the other two were not.

### Drugs

For the *in vitro* assays, antimicrobials were used as standard laboratory powders (Sigma-Aldrich, Madrid, Spain). For *in vivo* experiments, clinical formulations of antimicrobials were used: fosfomycin intravenous 1 g; (*Laboratorios* ERN, S.A; Barcelona, Spain), Amikacin Normon 125 mg/ml (*Laboratorios* Normon, S.A; Madrid, Spain) and gentamicin, genta-gobens 80 mg, (*Laboratorios* Normon, S.A; Madrid, Spain).

### *In vitro* Studies

#### Antimicrobial Susceptibility Testing

The minimal inhibitory concentrations (MIC) values were tested. MICs of fosfomycin, gentamicin and amikacin were determined by broth microdilution method as recommended by the Clinical Laboratory Standard Institute (CLSI) (19), using Mueller Hinton broth II (MHB) (Becton Dickinson & Co., Sparks, MD, United States) supplemented or not with 25 mg/L of glucose-6-phosphate (G-6-P) (Sigma-Aldrich, Madrid, Spain) and agar dilution method using Mueller Hinton agar supplemented with G-6-P for fosfomycin. MIC of fosfomycin was also determined by the broth microdilution method using MHB supplemented with G-6-P. MIC results were interpreted according to the European Committee on Antimicrobial Susceptibility Testing (EUCAST) (http://www.eucast.org/clinical_breakpoints/) breakpoints for all antibiotics (20). Studies were performed in triplicate to ensure reproducibility.

#### Time-Kill Curves

Time-kill methodology was used to study the *in vitro* interactions between fosfomycin plus amikacin and gentamicin. The antibiotic concentrations used for susceptible strains corresponded to the value of their MIC obtained by the broth microdilution method, whereas the concentrations used for resistant strains were those of the susceptibility breakpoints recommended by EUCAST. Experiments were carried out in MHB supplemented with G-6-P with a starting inoculum of 1 × 10^6^ cfu/mL and the drugs alone and in combination. Tubes were incubated at 37°C, with shaking and samples were taken at 0, 1 3, 6, and 24 h, serially diluted, plated (Eddy Jet, IUL S.A., Barcelona, Spain) and incubated at 37°C (16, 21). Bacterial colonies were counted after 24 h using an automatic colony counter (Flash & Go, IUL S.A., Barcelona, Spain). Experiments were performed at least three times on separate occasions. Bactericidal activities of single drugs or combination were defined as a decrease ≥ 3 log_10_ cfu/mL from the starting inoculum, bacteriostatic effect was defined as no change respect to the initial bacterial concentration during the 24 h. Synergy was defined as a decrease ≥ 2 log_10_ cfu/mL for the drugs combination compared with the most active single agent (22).

#### Animals

Immunocompetent C57BL/6J female mice weighing 16–20 g (7–9 weeks old) were used (Production and Experimentation Animal Center, University of Seville, Seville, Spain). Animals had a sanitary status of murine pathogen free and were assessed for genetic authenticity. Mice were housed in an individually ventilated cage system under specific pathogen-free conditions, with water and food *ad libitum*. This study was carried out in strict accordance with the recommendations in the Guide for the Care and Use of Laboratory Animals (23). *In vivo* experiments were approved by the Committee on the Ethics of Animal Experiments of the University Hospital Virgen Macarena, Seville, Spain (CI 1961). Procedures were performed under sodium thiopental (B. Braun Medical S.A., Spain) anesthesia, and all efforts were made to minimize suffering.

#### Pharmacokinetic/Pharmacodynamic Analysis

Serum antibiotic concentrations were determined in groups of healthy mice after a single intraperitoneal administration of fosfomycin (500 mg/kg), amikacin (15 mg/kg) or gentamicin (5 mg/kg). In sets of three animals and at 5, 10, 15, 30, 60, 90, 120, 240, 480, and 1,440 min after the administration of each antibiotic, blood samples were obtained from anesthetized mice from the periorbital plexus. Blood samples were immediately centrifuged at 4,500 rpm for 15 min at 4°C, and serum samples were stored at −80°C until its analysis. Serum concentrations of fosfomycin, amikacin and gentamicin were measured using a HPLC-tandem mass spectrometry (LC-MS/MS) (24). Measurement of the fosfomycin, amikacin and gentamicin binding to mice plasma proteins was also performed (25).

The maximum concentration of drug in serum (C_max_), elimination half-life (t_1/2_), Area Under the concentration-time Curve from 0 to 24 h (AUC_0−24_), free AUC_0−24_ (*f* AUC_0−24_), AUC_0−24_/MIC ratio, and *f* AUC_0−24_/MIC ratio were calculated using the PKSOLVER program (26). The pharmacodynamic parameters used to assure the efficacy of each antimicrobial C_max_/MIC for amikacin and gentamicin (27) and for fosfomycin this parameter is still not elucidated (28).

#### Murine Experimental Models

A previously described murine peritoneal sepsis model was used (22). Briefly, groups of C57BL/6J female mice were infected by intraperitoneal (ip) inoculation of 0.5 mL the Minimal Lethal Dose (MLD) of each strain. The inoculum concentrations inoculated were (log_10_ CFU/ml): 8.97 for VIM-1, 8.94 for VIM-1/DHA-1, 9.35 for OXA 48 plus CTX-M-15, and 8.38 for KPc29 KPC3/TEM-1 and SHV-11. Antimicrobial treatments were initiated 4 h post-inoculation and lasted 72 h. Mice were randomly included into six different therapeutic groups: (i) controls (untreated), (ii) fosfomycin, 500 mg/kg/8 h/ip (1.5 g/Kg/day), (iii) amikacin, 10 mg/kg/12 h/ip (100 mg/Kg/day), (iv) gentamicin, 5 mg/kg/12 h/ip (10 mg/Kg/day), (v) fosfomycin plus amikacin, for three of the strains: VIM-1 producer, VIM-1/DHA-1 producer, and OXA 48 plus CTX M 15 producer, (vi) fosfomycin plus gentamicin, for the KPC-3 TEM 1 plus SHV-11 producer strain. Antimicrobial dosages were chosen after PK/PD analysis to achieve the drugs optimal therapeutic regimen. Antimicrobials therapy lasted 72 h.

Immediately after mice death or sacrificed (sodium thiopental, ip) when the 72 h treatment was completed, aseptic thoracotomies were carried out, and blood samples were obtained for qualitative blood cultures; results were expressed as positive (≥1 cfu present in the plate) or negative. Spleens were aseptically extracted, weighed and homogenized in sterile saline (Stomacher 80; Tekmar Co., Cincinnati, OH, USA) before quantitative cultures (log_10_ cfu/g) in Columbia agar with 5% sheep blood plates (22).

Previous to the experimental model, in order to discard the toxicity of the treatments, groups of 5 healthy mice received the same dosages of antimicrobials alone or in combination, during 72 h, and were observed for any adverse reaction or body weight loss during 7 days.

### Statistical Analysis

Mortality and positive blood cultures were expressed as percentages. Bacterial spleen concentrations (Log_10_ CFU/g) were expressed as means ± SD. Differences in bacterial concentrations in spleen were compared by analysis of variance (ANOVA) and the Dunnet and Tukey *post-hoc*-tests. Mortality and blood sterility rates between groups were compared by use of the two-tailed Fisher's-test. A *P* < 0.05 value was considered as statistically significant. The SPSS v22.0 was used (SPSS Inc., Chicago, IL, USA).

## Results

### *In vitro* Results

#### Antimicrobial Susceptibilities

The MICs of fosfomycin, amikacin and gentamicin for the four clinical strains by the broth microdilution method are shown in Table 1. VIM-1-, OXA-48 plus CTX-M-15-, and KPC-3-producing strains were resistant to fosfomycin, whereas by the dilution in agar method only KPC-3 producer was resistant. Only the KPC-3-producing strain was resistant to amikacin, and the VIM-1-producer strain to gentamicin. No differences in MIC values were obtained when MHB was supplemented with G-6-P.

**Table 1:** MICs for the carbapenem-resistant *Klebsiella pneumoniae* strains used in the studies.

**Strains**	**MIC (mg/L) (SR)**		
	**Fosfomycin**	**Amikacin**	**Gentamicin**
VIM-1	8/64 (S/R)^*^	1 (S)	4 (R)
VIM-1/DHA-1	8/32 (S)^*^	4 (S)	2 (S)
OXA-48 plus CTX-M-15	16/>64 (S/R)^*^	1 (S)	0.5 (S)
KPC-3 (TEM-1 and SHV-11)	>64/>64 (R)^*^	>32 (R)	2 (S)

* *MIC values by dilution in agar dilution method/microdilution method*.

*S, susceptible; R, resistant*.

*For fosfomycin, susceptible ≤ 32 mg/L and resistant was > 32 mg/L. For amikacin, susceptible was ≤ 8 mg/L and resistant was > 8 mg/L. For gentamicin, susceptible was ≤ 2 mg/L and resistant was > 2 mg/L*.

#### Time-Kill Curves

The results are shown in Table 2. Fosfomycin plus amikacin was bactericidal and synergistic against VIM-1-, VIM-1 plus DHA-1-, and OXA-48 plus CTX-M-15-producing strains. Fosfomycin plus gentamicin showed synergistic activity only against the KPC-3 producer.

**Table 2:** Time-kill curves for fosfomycin (FOF) plus amikacin (AMK) and fosfomycin (FOF) plus gentamicin (GEN) against four clinical strains of carbapenemase producing strains.

**Strains**	**Effect of the antibiotic combination (grow time[s][h])**	
	**FOF + AK**	**FOF + GEN**
VIM-1	Synergy (*) and bactericidal effect	Indifference
VIM-1/DHA-1	Synergy (*) and bactericidal effect	Indifference
OXA-48 plus CTX-M-15	Synergy (**) and bactericidal effect	Indifference
KPC-3 (TEM-1 and SHV-11)	Indifference	Synergy (*)

*FOF, fosfomycin; AK, amikacin; GEN, gentamicin*.

* at 24 h;

** *at 6 and 24 h*.

#### Pharmacokinetics and Pharmacodynamics

Pharmacokinetic parameters of each antimicrobial are shown in Table 3. Pharmacodynamics profiles are shown in Table 4.

**Table 3:** Pharmacokinetic profiles of fosfomycin, amikacin and gentamicin in mice serum.

**Antimicrobial [dose (mg/kg), route of administration]**	**Drug**	**C_**max**_ (mg/L)**	**t_**1/2**_ (h)**	**AUC_**0−24**_ (mg·h/L)**
FOF (500, ip.)	*t*FOF	1354.09	1.07	2695.35
	*f*FOF	1340.55	1.09	2668.35
AK (15, ip.)	*t*AK	35.93	0.27	85.24
	*f*AK	27.93	0.27	63.06
GEN (5, ip.)	*t*GEN	21.03	0.43	40.23
	*f*GEN	14.72	0.37	28.16

*FOF, fosfomicin; AK, amikacin; GEN, gentamicin; tFOF, total fosfomicin; fFOF, free fosfomicin; tAK, total amikacin; fAK, free amikacin; tGEN, total gentamicin; fGEN, free gentamicim; ip., intraperitoneal; C_max_, maximum concentration of drug in serum; t_1/2_, half-life; AUC_0−24_, area under the concentration-time curve from 0-24 h; fAUC_0−24_, free AUC_0−24_*.

**Table 4:** *In vivo* efficacy and pharmacodynamics of fosfomycin, amikacin and gentamicin, alone and in combination, for the experimental peritoneal sepsis model.

**Strains**	**Treatment group**	**n**	**Spleen log_**10**_ cfu/g (mean ± SD)**	**Bacteremia (%)**	**Mortality (%)**	**C_**max**_/MIC**	***f*C_**max**_/MIC**	**AUC_**0−24**_/MIC**	***f*AUC_**0−24**_/MIC**
VIM-1	CTL	10	8.98 ± 0.46	100	100	ND	ND	ND	ND
	FOF	13	**7.23** **±** **0.48**^**a**^	100	100	21.16	20.95	42.11	41.69
	AK	15	**5.36** **±** **2.46**^**a**^	**40**^**a**^**,** ^**b**^	80	35.93	27.93	85.24	63.06
	FOF+AK	14	**6.62** **±** **0.38**^**a**^^,^ ^**b**^	**28.57**^**a**^**,** ^**b**^	100	ND	ND	ND	ND
VIM-1/DHA-1	CTL	10	9.46 ± 0.32	100	100	ND	ND	ND	ND
	FOF	15	8.64 ± 0.94	100	93.3	42.32	41.89	84.23	83.39
	AK	15	8.60 ± 1.48	100	86.67	8.98	6.98	21.31	15.77
	FOF+AK	15	8.43 ± 1.36	100	93.33	ND	ND	ND	ND
OXA-48 plus CTX-M-15	CTL	10	9.56 ± 0.47	100	100	ND	ND	ND	ND
	FOF	15	**8.05** **±** **0.30**^**a**^	100	100	21.16	20.95	42.11	41.69
	AK	14	**7.83** **±** **1.07**^**a**^	78.57	78.57	35.93	27.93	85.24	63.06
	FOF+AK	15	**7.74** **±** **0.67**^**a**^	80	100	ND	ND	ND	ND
KPC-3 (TEM-1 and SHV-11)	CTL	10	10.19 ± 0.29	100	100	ND	ND	ND	ND
	FOF	15	9.86 ± 0.24	100	100	21.16	20.95	42.11	41.69
	GEN	15	9.14 ± 2.39	100	86.67	10.06	7.36	20.12	14.08
	FOF+GEN	15	**9.02** **±** **0.68**^**a**^**,** ^**b**^	100	93.33	ND	ND	ND	ND

*CTL, control (no antimicrobial treatment); FOF, fosfomycin; AK, amikacin; GEN, gentamicin*.

a *P ≤ 0.05 compared to CTL groups*.

b *P ≤ 0.05 compared to FOF groups*.

*ND, no determined. VIM-1 and OXA-48 plus CTX-M-15 strains, AMK MIC: 1 mg/L; VIM-1 (DHA-1) AMK MIC: 4 mg/L; KPC-3 (TEM-1 and SHV-11) GEN MIC: 2 mg/L*.

*Bold tries to remark the treatments that were significantly better than controls and other treatments*.

#### *In vivo* Results: Peritoneal Sepsis Model

The efficacies of the antimicrobials are shown in Table 4. Mortality, bacterial clearance on spleen and bacteremia, are analyzed immediately after the death of mice or at the end of the experiment (72 h of treatment).

#### Mortality

Mortality in all control groups (non-treated) was 100% within the first 24 h post-infection. Antimicrobials alone or in combination did not significantly reduce mortality against any of the strains.

#### Bacterial Clearance From Spleen

Fosfomycin alone improved significantly the clearance of bacteria from spleen (CFU/g of tissue) compared with the control groups in mice infected with either VIM-1 (7.23 ± 0.48 vs. 8.98 ± 0.46) or OXA-48 plus CTX-M-15 (8.05 ± 0.30 vs. 9.56 ± 0.47). Amikacin alone was also better than controls in reducing bacterial concentration in spleen against VIM-1 or OXA-48 plus CTX-M-15 strains (5.36 ± 2.46 vs. 8.98 ± 0.46 and 7.83 ± 1.07 vs. 9.56 ± 0.47, respectively). Moreover, the combination of fosfomycin plus amikacin reduced the bacterial concentration compared with the controls for the strains, producing VIM-1 or OXA-48 plus CTX-M-15 (6.62 ± 0.38 vs. 8.98 ± 0.46 and 7.74 ± 0.67 vs. 9.56 ± 0.47, respectively) and fosfomycin plus gentamicin for the strain producing KPC-3 (9.02 ± 0.68 vs. 10.19 ± 0.29). Fosfomycin plus amikacin against the VIM-1 strain reduced the spleen bacterial concentration compared to fosfomycin alone (6.62 ± 0.38 vs. 7.23 ± 0.48). None of the antimicrobials tested alone or in combination reduced the bacterial concentration in spleen against the strain producing VIM-1 (DHA-1).

#### Bacteremia

Amikacin alone and in combination with fosfomycin showed a significant reduction sterilizing blood cultures of mice infected with the VIM-1 producer (40 and 28.57% vs. 100%, respectively).

## Discussion

The results of this study show that the combination of fosfomycin plus aminoglycosides (amikacin and gentamycin) shows efficacy in terms of bacterial clearance from tissue in animals infected with the strains VIM-1, OXA-48 plus CTX-M-15 and KPC-3 producers. Bacteraemia was only significantly reduced against the VIM-1 producer strain with fosfomycin plus amikacin. None of the treatments tested improved mortality compared to those from control groups for any of the studied strains. Finally, there was no significant efficacy in terms of bacterial clearance either from tissue and blood or in the survival in the animals infected with the strain producing VIM-1 (DHA-1). The synergistic activity found between fosfomycin and amikacin is in accordance with other *in vitro* studies that found a synergistic and bactericidal effect with this combination against fosfomycin susceptible clinical KPC-producing *K. pneumoniae* isolates, regardless the bacteria susceptibility to amikacin (15).

Fosfomycin dosage used in this study 500 mg/kg every 8 h was chosen because it produces a mean peak serum fosfomycin concentration of 1354.09 ± 217.99 mg/L and a mean AUC_0−24_ of 2695.35 ± mg^*^h/L, above to those obtained in humans after a 4 h infusion of 4 g every 8 h (peak of 123 + 16 mg/L; AUC0–24 of 600 mg^*^h/L) and above dosages proven to be effective in several murine experimental studies using, KPC-3- or OXA-48-producing *Escherichia coli* (29), *Enterococcus faecalis* (30). Moreover, with this dose we achieved the PK/PD index that best predicted efficacy for *K. pneumoniae* strains base on model fit and regression analysis, an unbound AUC_0−14_/MIC ratio of 41.69 or 83.39, depending on the strains (31). Nevertheless, fosfomycin monotherapy was only effective in reducing tissue concentrations against VIM-1- and OXA-48 plus CTX-M-15-producing strains, but not with the other two tested strains, not even with the fosfomycin susceptible VIM-1/DHA-1-producing strain. These results could be in the line of the described uncertainty as whether fosfomycin monotherapy is efficacious in the treatment of systemic infections other than complicated urinary tract infection and/or acute pyelonephritis (32).

Although the pharmacokinetic/pharmacodynamic target C_max_/MIC ≥ 8 (33) was achieved for all the tested strains, the activity of aminoglycosides (amikacin and gentamicin) in monotherapy was also limited, reducing amikacin only bacterial spleen concentration compared to control groups in mice infected with VIM-1 or OXA-48 plus CTX-M-15 strains, for which the Cmax/MIC value for both strains was = 35.93, predictor of efficacy was achieved. Only in the case of animals infected with the VIM-1 producer strain, amikacin reduced the mortality compared to controls mice. Nevertheless, amikacin is only used alone to treat urinary tract infections (34).

Fosfomycin in combination with amikacin demonstrated *in vitro* a synergistic effect against the VIM-1-, VIM-1/DHA-1-, and OXA-48 plus CTX-M-15-producing strains, while the combination with gentamycin was the only with synergistic activity against KPC-3-producing strain. These results are in accordance with other studies showing synergy of this combination against KPC-producing *K. pneumoniae* strains. An *in vitro* study in KPC-producing K. pneumoniae found a synergistic and bactericidal effect with the combination of fosfomycin with amikacin against fosfomycin susceptible isolates, even when the causative bacteria are resistant to amikacin (15). Other *in vitro* studies have showed fosfomycin plus amikacin to have additive and synergistic effects against other pathogens such as *P. aeruginosa* and methicillin-resistant *Staphylococcus epidermidis* and IMP-8 metallo-β-lactamase-producing *K. oxytoca* (35, 36).

With regard to the *in vivo* efficacy of fosfomycin plus amikacin in the experimental murine peritoneal sepsis model, the combination was better than the control groups taking into account the bacterial clearance from spleen against the VIM-1 and the OXA-48 plus CTX-M-15 strain. Moreover, this combination reduced bacteremia compared both with control and fosfomycin groups against the VIM-1 strain. Nevertheless, and besides both antimicrobials achieving the pharmacodynamic values described as optimal for efficacy, *f* AUC_0−24_/MIC and C_max_/MIC, no activity is found against the VIM-1/DHA-1 strain, in contrast with the synergy observed in the time-kill studies. Also, and in accordance with the *in vitro* results fosfomycin plus gentamicin reduced significantly bacteria from spleen compared with control and fosfomycin groups against the KPC-3 strain. Finally, none of the combinations reduced the mortality against any of the tested strains. To our best knowledge, there are no experimental studies in which fosfomycin in combination with amikacin and gentamicin has been evaluated. There are experimental studies that have evaluated fosfomycin in combination with ceftazidime-avibactam combination against MDR *P. aeruginosa* finding this combination better than either drug alone (37). In other study, they evaluated in a surgical wound infection in mice the activity of fosfomycin in combination with rifampin and tigecycline against *Enterococcus faecium* and methicillin-resistant *Staphylococcus aureus* clinical isolates, finding the combination as an alternative treatment to control skin infection (38). Also, Berleur et al., found that fosfomycin plus temocillin reduced bacterial counts in a murine peritonitis model against *E. coli* strains producing KPC-3 or OXA-48-type carbapenemases (29).

In summary, our results showed that the fosfomycin plus aminoglycosides combination has a low and dissimilar efficacy in the treatment of severe infections, such as a peritoneal sepsis infection, caused by different KPC-KP strains, reducing only the tissue bacterial concentration against three of the strains and only decreasing bacteremia against the VIM-1 producer strain. Moreover, none of the combinations improved the survival in the infection by any of the KPC-KP strains. Also no activity was found in reducing tissue bacterial concentration or in decreasing bacteremia or mortality in the infection by VIM-1/DHA-1 strain. Because of the lack of available alternatives for these kinds of carbapenemase-producing strains, the fosfomycin plus aminoglycosides combination might be considered for further evaluation against other kind of infections. Overall, the results of the present study suggest that the efficacy of fosfomycin plus aminoglycosides depends on the class of carbapenemase produced by *K. pneumoniae*.

## Data Availability Statement

The raw data supporting the conclusions of this article will be made available by the authors, without undue reservation.

## Ethics Statement

*In vivo* experiments were approved by the Committee on the Ethics of Animal Experiments of the University Hospital Virgen Macarena, Seville, Spain (CI 1961).

## Author Contributions

MP-I planned and coordinated the experiments, analyzed the results, and written the manuscript. GL-H and TC-C had performed the *in vitro* and *in vivo* experiments. CD and JdP had performed the antibiotic concentrations studies by HPLC tandem mass spectrometry (LC-MS/MS). JR-B, ÁP, and JP had reviewed the manuscript and the experiments. MC obtained the funds to perform the studies and wrote the project, contributed to the performance of the *in vitro* experiment, and reviewed the results and the manuscript. All authors contributed to the article and approved the submitted version.

## Conflict of Interest

The authors declare that the research was conducted in the absence of any commercial or financial relationships that could be construed as a potential conflict of interest.
